# Association between intensity or accumulating pattern of physical activity and executive function in community-dwelling older adults: A cross-sectional study with compositional data analysis

**DOI:** 10.3389/fnhum.2022.1018087

**Published:** 2023-01-25

**Authors:** Kazuki Hyodo, Naruki Kitano, Aiko Ueno, Daisuke Yamaguchi, Yuya Watanabe, Takayuki Noda, Sumiyo Nishida, Yuko Kai, Takashi Arao

**Affiliations:** Physical Fitness Research Institute, Meiji Yasuda Life Foundation of Health and Welfare, Tokyo, Japan

**Keywords:** accelerometer, Stroop, inhibition, working memory, cognitive flexibility, cognitive function, task-switching, N-back

## Abstract

**Objective:**

Previous studies have suggested a positive association between physical activity (PA) and executive function in older adults. However, they did not adequately consider the compositional nature of daily time use and accumulated PA patterns. Therefore, this study aimed to examine the association between intensity or accumulated PA patterns and executive functions (inhibitory control, working memory, and cognitive flexibility) in community-dwelling older adults, considering the interaction of daily time spent in PA, sedentary behavior (SB), and sleep.

**Method:**

This cross-sectional study used baseline data from a randomized controlled trial on the effect of exercise on cognitive function conducted between 2021 and 2022. Data from 76 community-dwelling older adults were used in the analysis. The time spent in PA and SB was assessed using an accelerometer, and sleep duration was self-reported. The Stroop task (inhibitory control), N-back task (working memory), and task-switching task (cognitive flexibility) were conducted to evaluate the subcomponents of executive function. Considering various potential confounders, compositional multiple linear regression analysis and compositional isotemporal substitution were performed to examine the association of PA with executive function and to estimate predicted changes in executive function in response to the hypothetical time-reallocation of movement behaviors, respectively.

**Results:**

A longer time spent in light-intensity PA (LPA), relative to remaining behaviors, was associated with better Stroop task performance. Moreover, this association was stronger in LPA lasting longer than 10 min than in sporadic LPA. Additionally, theoretical 30 min/day time reallocation from SB or sleep to LPA was associated with better Stroop task performance (corresponding to approximately a 5%−10% increase). On the other hand, no significant associations of time spent in moderate- to vigorous-intensity PA with any subcomponents of executive function were observed.

**Conclusion:**

LPA was positively associated with inhibitory control, and this association was stronger in bouts of LPA than in sporadic LPA. Moreover, reducing the time spent in SB or sleep and increasing the time spent in LPA, especially long-bout LPA, could be important measures for managing inhibitory control in late life. Future large longitudinal and intervention studies are needed to confirm these associations and reveal the causality and underlying mechanisms.

## 1. Introduction

Executive function is broadly defined as a complex higher-order cognitive processes, including inhibitory control, working memory, and cognitive flexibility, which control behaviors toward a goal (Miyake et al., 2000). Executive function is necessary for independent living later in life (Vaughan and Giovanello, 2010; Nguyen et al., 2019); however, this function shows age-related decline (Park et al., 2002; Reuter-Lorenz et al., 2021). Therefore, preserving this function in later life is important for maintaining the quality of life in older adults. Daily physical activity (PA), including exercise, has attracted attention as a modifiable lifestyle factor for maintaining executive function in late adulthood (Mellow et al., 2022). This is supported by the findings that exercise induces molecular, cellular, and structural changes in the brain (El-Sayes et al., 2019). Although most previous studies have examined the effect of moderate- to vigorous-intensity exercise on executive function (Chen et al., 2020), several systematic reviews and meta-analysis have reported the possible beneficial effect of light-intensity exercise (Gomes-Osman et al., 2018; Sanders et al., 2019). Moreover, daily PA, mainly non-exercise activity, is also reported to be associated with higher executive function. Specifically, several observational studies have reported that light-intensity PA (LPA) or moderate- to vigorous-intensity PA (MVPA) in daily living are associated with better executive functions, such as inhibitory control, working memory, and cognitive flexibility (Wilbur et al., 2012; Johnson et al., 2016; Umegaki et al., 2018; Spartano et al., 2019; Gerten et al., 2021; Gothe, 2021).

However, these studies have some methodological limitations. First, previous studies have not sufficiently considered the co-dependent nature of daily time use. Since a day is fixed at 24 h, consisting of PA, sedentary behavior (SB), and sleep, whenever the time for one behavior (e.g., PA) is increased, the time spent on other behaviors (e.g., SB or sleep) essentially decreases. Since SB (Steinberg et al., 2015) and sleep (Lo et al., 2016; Richards et al., 2017) are also considered determinants of executive function, the relationship between PA and executive function is likely to differ depending on the behavior used to engage in PA (Chastin et al., 2015; Dumuid et al., 2018). Only two cross-sectional studies have attempted to address this issue and examined the association between time substitution from SB or sleep to PA and executive function in older adults (Fanning et al., 2017; Wei et al., 2021). However, these studies treated the time spent on movement behaviors as an absolute measure. In other words, these studies violate the compositional nature of daily time use and have potentially misleading results (Dumuid et al., 2019). An alternative approach is to use compositional data analysis (CoDA). In CoDA, data on the daily time spent on PA, SB, and sleep as compositions allows us to address the co-dependent nature appropriately. This statistical technique has traditionally been used in various fields, including geochemistry, nutrition, and politics (Chastin et al., 2015). Since Chastin et al. proposed the CoDA as a technique to analyze daily time use in 2015 (Chastin et al., 2015), many studies have examined the association between movement behaviors and health outcomes using this statistical approach (Janssen et al., 2020). In some cases, the results from conventional statistical models and the CoDA have been reported to be not meaningfully different (Biddle et al., 2018). However, the appropriateness of the CoDA approach in time-use data stands and, to examining the relationship between PA and health outcomes, it has been recommended to use this statistical technique (Chastin et al., 2015; Pedišić et al., 2017; Dumuid et al., 2018).

Second, these two studies failed to consider the PA accumulation pattern. Recently, it has been suggested that the association between PA and executive function may depend on the bout length of PA. Experimental studies suggest that PA's positive effect on executive function in older adults is meaningful only if it lasts for more than a few minutes (Chang et al., 2019a,b). Conversely, two cross-sectional studies have reported that even short PA bout lengths (e.g., ≤ 5 or 10 min) are associated with better inhibitory control and working memory (Peven et al., 2018; Wanigatunga et al., 2018). However, the inconsistent results of previous studies suggest a lack of evidence on these associations, and the role of PA bout length in relation to executive function is unclear.

Research that overcomes these limitations would help clarify “which behaviors need to be reduced and which type of PA increased instead in daily living.” In addition, this finding contributes to the development of strategies for managing executive function in later life. Therefore, this cross-sectional study aimed to examine the associations between accelerometer-measured PA and the range of executive functions in older adults, considering the co-dependent nature of daily time use using the CoDA. Furthermore, this study aimed to estimate the association between time reallocation from SB or sleep to PA and executive function in older adults.

## 2. Materials and methods

### 2.1. Participants and procedure

This cross-sectional study used baseline data from an online exercise intervention. We conducted two exercise interventions between September and November 2021 and January and April 2022 (trial Registration: UMIN-CTR UMIN000044758). Baseline accelerometer data were collected in August 2021 and January 2022. Participants were community-dwelling older adults in Hachioji city, Tokyo, Japan, recruited through flyers and word of mouth. A total of 102 older adults were enrolled in the intervention study. After receiving an explanation of the study, written informed consent was obtained from each participant before the study. Then, the participants answered questionnaires about their demographic data and health status and underwent the Mini-Mental State Examination (MMSE). Afterward, the participants took cognitive tasks individually in a single quiet room. They were then given an accelerometer and instructed to wear it for ≥10 consecutive days. The inclusion criteria for this study were participants who (1) were > 60 years old, (2) did not have severe cognitive impairment (MMSE score ≥21), (3) did not have a history of central nervous diseases such as stroke or Parkinson's disease, and (4) were not restricted from exercising by a physician. Consequently, 89 older adults met our inclusion criteria. The Ethics Committee of the Physical Fitness Research Institute of Meiji Yasuda Life Foundation of Health and Welfare approved this study (approval number: 2021-0001).

### 2.2. Movement behavior

A triaxial accelerometer (Active style Pro HJA750- C; Omron Healthcare, Kyoto, Japan) was used to assess participants' PA and SB. The participants were instructed to wear the accelerometer on their hips for at least 10 days during awake hours. This accelerometer has been validated, and its measurement accuracy is comparable to that of other devices widely used in Western countries (Murakami et al., 2016; Kurita et al., 2017). The epoch length was set at 60 s, and the estimated metabolic equivalents (METs) were obtained using developer-provided software. Nonwear time was defined as an interval of 60 consecutive minutes with activity counts below the detection limit. The day the participant wore the device for ≥10 h was considered a valid day (Tudor-Locke et al., 2012). Participants with ≥4 valid days of data collection were included in the analysis. Each 60 s epoch was classified as SB ( ≤ 1.5 METs), LPA (1.6–2.9 METs), or MVPA (≥3.0 METs) (Haskell et al., 2007; Pate et al., 2008). We also evaluated bouted LPA (BLPA, ≥10 consecutive minutes with an allowance for interruptions of up to 2 min below the LPA threshold) and bouted MVPA (BMVPA, ≥10 consecutive minutes with an allowance for interruptions of up to 2 min below the MVPA threshold). This 2-min allowance accommodates real-world activity patterns (e.g., stopping at crosswalks while walking or jogging or brief rests during sports) (Saint-Maurice et al., 2018). The time spent on each of these behaviors was aggregated per day and averaged over all valid days. We also assessed participants' nocturnal sleep duration using an item on sleep duration from the Japanese version of the Pittsburgh Sleep Quality Index (Doi, 1998). Based on previous studies (Del Pozo Cruz et al., 2020; McGregor et al., 2020; Tsunoda et al., 2021), in this study, data from the questionnaire (i.e., sleep) and the device (i.e., PA and SB) were integrated and standardized into a 24-h for all participants' behaviors. Specifically, we first subtract the sleep duration from 24 h to calculate the theoretical waking hours. Then, based on the proportion of PA and SB accounted for the accelerometer wearing time, we re-distributed the theoretical wake hours to the respective activity time.

### 2.3. Executive function

In this study, we used computer-based Stroop, N-back, and task-switching tasks to assess inhibitory control, working memory, and cognitive flexibility, respectively. Stimuli were presented using the Psychopy3 software (Peirce et al., 2019). The participants performed these tasks in a counterbalanced random order in a single quiet room. Before measuring each cognitive task performance, participants practiced the task to learn the rules.

#### 2.3.1. Stroop task

Inhibitory control was assessed using a computer-based color-word matching Stroop task (Zysset et al., 2001; Hyodo et al., 2016). Two rows of letters were presented on a computer screen. The participants were required to determine whether the color of the words or letters in the top row matched the name of the color printed in the bottom row. They had to press the N key with their left index finger if it matched or the C key with their right index finger if it did not match as quickly and accurately as possible. The task comprised neutral and incongruent conditions, which consisted of 20 trials each. For neutral conditions, the letter sequence “XXXX” was displayed in the top row in red, green, blue, or yellow, and the hiragana (Japanese alphabet) words “あか” (red), “みどり” (green), “あお” (blue), or “きいろ” (yellow) was displayed in the bottom row in black. For incongruent conditions, the top row contained the word “あか,” “みどり,” “あお,” or “きいろ” displayed in an incongruent color (e.g., “あお” in red). Each trial lasted 3 s, with the top row displayed 350 ms ahead, followed by both rows for 2,650 ms. The top row appeared earlier than the bottom row to achieve sequential visual attention. In the task, 40 trials (20 neutral and 20 incongruent) were presented randomly. The number of trials in which the C key was the correct answer and the number of trials in which the N key was the correct answer were the same. The inter-stimulus interval (ISI) was 9, 10, or 11 s with a fixation cross presented on the center of the screen. Percent of correct responses (accuracy, AC) and mean reaction time of corrected responses (RT) for the neutral and incongruent conditions were measured.

#### 2.3.2. N-back task

The N-back task measured working memory ability (Kirchner, 1958). The N-back task consisted of the 0-back (no working memory load) and 2-back (high working memory load) conditions. In each condition, a single hiragana character from “あ” to “こ” (10 alphabetical letters in Japanese) was preudo-randomly presented on the screen. For the 0-back condition, they were required to press the “N” key if the letter displayed on the screen was “あ,” and the “C” key if it was not “あ.” For the 2-back condition, they were instructed to press the “N” key if the letter shown on the screen was the same as the one presented two trials before and the “C” key if it differed from the one presented two trials before. The participants completed a total of four blocks with two blocks each of 0-back and 2-back conditions. Inter-block interval were 45 s (blanks for 30 s, explanation of the next block for 10 s, and fixation crosses for 5 s). The order of the four blocks was randomly counterbalanced. Each block consisted of 20 trials, of which five were correct for the N key. Each trial consisted of a 500-ms stimulus followed by a 2,500-ms ISI. The participants were required to press either the “C” or “N” keys as quickly and correctly as possible. AC and correct RT for the 0-back and 2-back conditions were measured.

#### 2.3.3. Task-switching task

To measure cognitive flexibility, participants conducted a color-shape task-switching paradigm (Hakun et al., 2015; Zhu et al., 2015). For this task, there were two possible shapes (circle or square) in two possible colors (red or blue). Participants were instructed to press either the “C” or “N” key to respond to each stimulus based on the instruction cue [either the hiragana word “いろ” (color) or “かたち” (shape)] appeared on the screen at the beginning of each trial. When the instruction cue was “いろ” (color), participants had to press the “C” key if the following stimulus (circle or square) appeared in red, and the “N” key if it appeared in blue. When the instruction cue was “かたち” (shape), the correct response was to press the “C” key if the following stimulus was a circle and the “N” key if it was a square. The duration was 3 s for each trial, which consisted of 200 ms of a fixation cross, 150 ms of instruction cue, and 2,650 ms of stimulus.

This task consisted of four blocks: two blocks (color block and shape block) in the single condition and two blocks in the mixed condition. Each block consisted of 20 trials. In the single condition block, the instruction cue was the same for all trials in each block (“いろ” for the color block and “かたち” for the shape block). Thus, participants judged whether the stimulus color was red or blue in the color block and whether the shape was circle or square in the shape block. For the mixed condition block, “shape” and “color” trials were pseudo-randomly presented with an equal number of repeating/switching in consecutive trials. The order of the blocks was fixed as participants completed the task in the following order: color block, shape block, and two mixed blocks. The inter-block interval was 45 s (blanks for 30 s, explanation of the next block for 10 s, and fixation crosses for 5 s). The participants were instructed to press the response key based on the instruction cue as quickly and correctly as possible. The AC and correct RT in each condition were measured.

#### 2.3.4. Data analysis

Considering the speed-accuracy trade-off in each cognitive task, the RT and AC were converted to the balance integration score (BIS) and used as the dependent variable in the analysis (Liesefeld and Janczyk, 2019). The BIS was calculated by subtracting the standardized RT from the standardized AC as in the following equation:


$$\begin{array}{l} BIS\;=\;z\left(AC\right)-z\left(RT\right) \end{array}$$

The standardized AC and RT were obtained using the mean and standard deviation of the participants for each task. Standardization was done by including all conditions for each task to avoid all conditions having the same value of 0.

Using this BIS, a single measure of executive function (inhibitory control, working memory, and cognitive flexibility) was obtained for each task and used as outcomes. As an evaluation of Inhibitory control, we calculated the BIS of Stroop interference (BIS of incongruent conditions – BIS of neutral conditions) in the Stroop task. We calculated the BIS of the 2-back condition to evaluate working memory in the N-back task. To evaluate cognitive flexibility, we used the BIS of global switching cost (Kray and Lindenberger, 2000), calculated by subtracting the BIS of the single condition from the BIS of the mixed condition. A higher BIS indicated better task performance. RT and AC, which were used to calculate BIS, were used as outcome measures in the sensitivity analysis.

### 2.4. Other variables

Potential confounders were selected based on *prior* clinical knowledge and previous studies (Fanning et al., 2017; Umegaki et al., 2018; Spartano et al., 2019; Gerten et al., 2021). These variables included age, sex, years of education, subjective economic status, living arrangement, social participation, depressive symptoms, and clinical history of hypertension, diabetes, and heart disease. In addition, body mass index (BMI), alcohol consumption, and smoking habits were investigated to identify the participant characteristics. Data were collected using self-administered questionnaires, except for BMI. The frequency of participation in volunteer groups, hobby groups, senior citizen clubs, neighborhood associations, learning/education circles, and paid work was assessed. Participants responded to each question with one of five options: never (1 point), 1–3 days/month (2 points), 1 day/week (3 points), 2–3 days/week (4 points), or ≤ 4 days/week (5 points). In the analysis, we used the sum of the scores assigned to each response to indicate social participation frequency. Depressive symptoms were assessed using the short version of the Geriatric Depression Scale. Older adults who scored >5 points were defined as having mild depressive symptoms (Yatomi, 1994). BMI was calculated from the measured body height and body weight.

### 2.5. Statistical analysis

All statistical analyses were performed using R 4.0.2 (R Foundation for Statistical Computing, Vienna, Austria) (R Core Team, 2020). Statistical significance was set at *p* < 0.05. We performed the CoDA as per previously reported (Chastin et al., 2015; Dumuid et al., 2019) using the packages “Compositions,” “robCompositions,” and “zCompositions.”

As 14 older adults did not engage in the BMVPA, a log-ratio expectation-maximization algorithm was used to impute these zeros (Palarea-Albaladejo and Martín-Fernández, 2015). In this study, we used the following two time-use compositions: a non-bouted model (SB, LPA, MVPA, and sleep) and a bouted model (SB, BLPA, non-BLPA, BMVPA, non-BMVPA, and sleep). Before the analysis, these time-use compositions were transformed to an isometric log-ratio (ilr) based on the pivot coordinate representation. As a result, a set of three (for a non-bouted model) or five (for a bouted model) ilr-coordinates were created where the first coordinate (ilr1) represented time spent in one behavior (e.g., LPA) relative to the remaining behaviors (e.g., MVPA, SB, and sleep). This procedure was repeated until we obtained ilr1s, representing the relative importance of LPA and MVPA (for the non-bouted model), BLPA, non-BLPA, BMVPA, and non-BMVPA (for the bouted model). The set of ilr-coordinates from each repetition was used as an independent variable in the following regression model. This means that the model also includes information on behaviors (e.g., MVPA, SB, and sleep) other than the PA (e.g., LPA), whose relative importance is indicated in the ilr1. Further details on the mathematical background of CoDA are described elsewhere (Chastin et al., 2015; Dumuid et al., 2018).

Compositional multiple linear regression with robust estimators was conducted to examine the associations between time spent in each PA relative to the remaining behaviors and executive function. Unstandardized regression coefficients and 95% confidence intervals (CIs) were calculated. The executive function indicator and PA (expressed as ilrs) were included as the dependent and independent variables in the model, respectively. We set two regression models. Model 1 included the following variables as covariates: age (continuous), sex (male/female), and years of education (continuous). Model 2 further added the following covariates to Model 1: subjective economic status (good, very good/poor, and very poor), living arrangement (living alone or with others), frequency of social participation (continuous), depressive symptoms ( ≤ 4 points/≥5 points), and clinical history of hypertension, diabetes, and heart disease (yes/no). Only results from ilr1s for each coordinate system are reported since results (i.e., coefficients) from ilr1s that contain information about the relative importance of a particular PA (e.g., LPA) to the remaining behaviors (e.g., SB, MVPA, and sleep) are the focus of this study. As a sensitivity analysis, we also performed a regression analysis with RT and AC as dependent variables instead of BIS.

In CoDA, regression coefficients of the time spent in PA, expressed in log-ratios, are difficult to interpret directly. Therefore, when a statistically significant association between time spent in PA and executive function indicators was confirmed from the above regression model, we conducted compositional isotemporal substitution, which estimates changes in executive function based on pairwise time reallocations, to interpret the results more meaningfully. In short, this statistical approach calculated predicted executive function performance using Model 2 of the above linear regression and the average of the participant's time-use composition (the compositional mean). Then, using a new composition (e.g., 10-min time-reallocation of SB to LPA), the predicted outcome value is again estimated. In the compositional isotemporal substitution, the difference between these two values considers as the theoretical change in executive function when re-allocating time between two (or more) behaviors (Dumuid et al., 2019). In this study, time from 10 to 30 min was reallocated from one movement behavior (e.g., SB) to PA (e.g., LPA) while maintaining the time spent in the remaining behaviors (e.g., MVPA and sleep) constant. Estimates were calculated in 10-min increments up to 30 min or the compositional mean of the behavior.

## 3. Results

### 3.1. Characteristics of study participants

Of the 89 eligible participants, 13 were excluded from analysis for the following reasons: invalid/missing data on the accelerometer (*n* = 1), refusal of the N-back task (*n* = 2), and incomplete data (*n* = 10). Finally, 76 older adults were included in the final analysis. The participants' mean age was 75.8 years (range: 63–88 years), 63% were female, the mean BMI was 22.4 kg/m^2^, and the mean education year was 13.0 years (Supplementary Table 1). Table 1 and Supplementary Table 2 show the descriptive statistics of the study participants' movement behaviors and each cognitive task performance. On average, participants wore an accelerometer for 905.1 min/day and spent 633.5 min for SB (44.0%), 391.9 min for LPA (27.2%), 36.5 min for MVPA (2.5%), and 378.1 min for sleep (26.3%), respectively. Regarding the accumulation pattern of PA, most LPA cases were BLPA, and conversely, most MVPA cases were sporadic.

**Table 1:** Characteristics of movement behaviors in the study participants.

**Accelerometer-assessed information**	***n* = 76**
	**Mean (SD)**
Wearing period, days	11.6 (2.1)
Wearing time, min/day	905.1 (78.4)
Time-use composition	Mean (%)^a^
**Without bouted PA, min/day**	
SB	633.5 (44.0)
LPA	391.9 (27.2)
MVPA	36.5 (2.5)
Sleep	378.1 (26.3)
**With bouted PA, min/day** ^b^	
SB	640.3 (44.5)
BLPA	278.9 (19.4)
Non-BLPA	106.9 (7.4)
BMVPA	6.9 (0.5)
Non-BMVPA	24.9 (1.7)
Sleep	382.2 (26.5)

SB, sedentary behavior; LPA, light-intensity physical activity; MVPA, moderate- to vigorous-intensity physical activity; BLPA, bouted LPA; BMVPA, bouted MVPA.

^a^In parentheses, the proportion (%) of each behavior to each composition is shown.

^b^In time-use compositions with bouted PA, zero count (0 min/day) in the bouted MVPA were imputed by using the log-ratio expectation-maximization algorithm (n = 14).

### 3.2. Associations of PA with executive function

**Figure 1:**
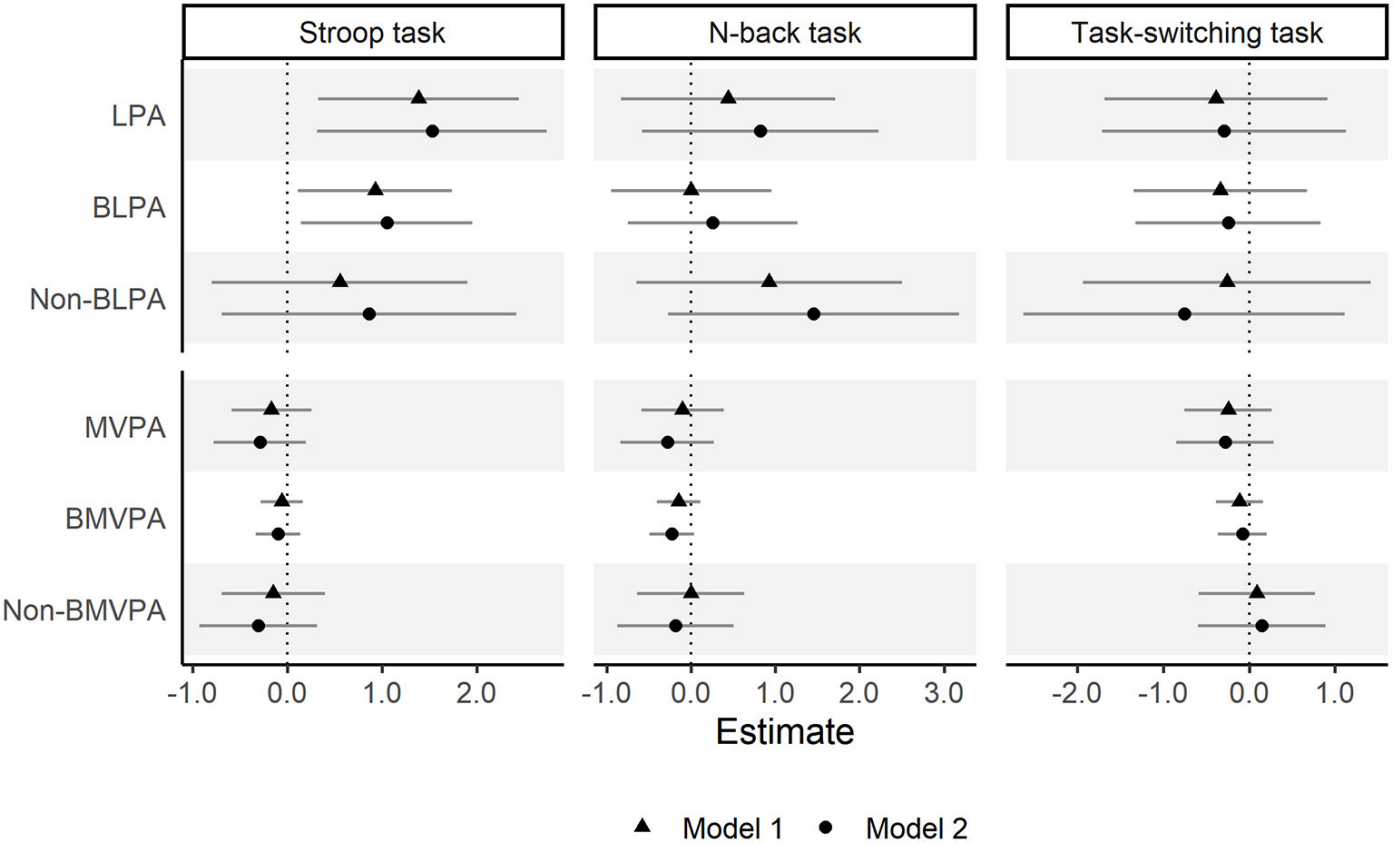
Associations between physical activity and executive function. Model 1 was adjusted for age, sex, and education year. Model 2 was further adjusted for subjective economic status, living arrangement, frequency of social participation, depressive symptoms, and clinical history (hypertension, diabetes, and heart disease). LPA, light-intensity physical activity; BLPA, bouted LPA; MVPA, moderate- to vigorous-intensity physical activity; BMVPA, bouted MVPA. Balance integration score of Stroop interference, 2-back condition, and global switching cost were used for evaluating Stroop task, N-back task, and task-switching task performance, respectively.

The compositional multiple linear regression analysis results are shown in Figure 1 (Supplementary Table 3). We confirmed that a longer time spent in LPA (relative to remaining behaviors) was associated with a better Stroop task performance (β = 1.53, 95% CI = 0.31, 2.74). More specifically, time spent in BLPA lasting longer than 10 min (relative to remaining behaviors) was associated with better Stroop task performance (β = 1.05, 95% CI = 0.14, 1.96), but non-BLPA did not. In contrast, none of the variables related to time spent in MVPA showed significant associations with Stroop task performance. In addition, we did not confirm any significant associations of LPA or MVPA with performance on the N-back or task-switching task.

Moreover, sensitivity analysis was performed using RT and AC as outcomes (Supplementary Figure 1). The direction and magnitude of the association between PA and task performance did not differ significantly from the BIS results.

**Figure 2:**
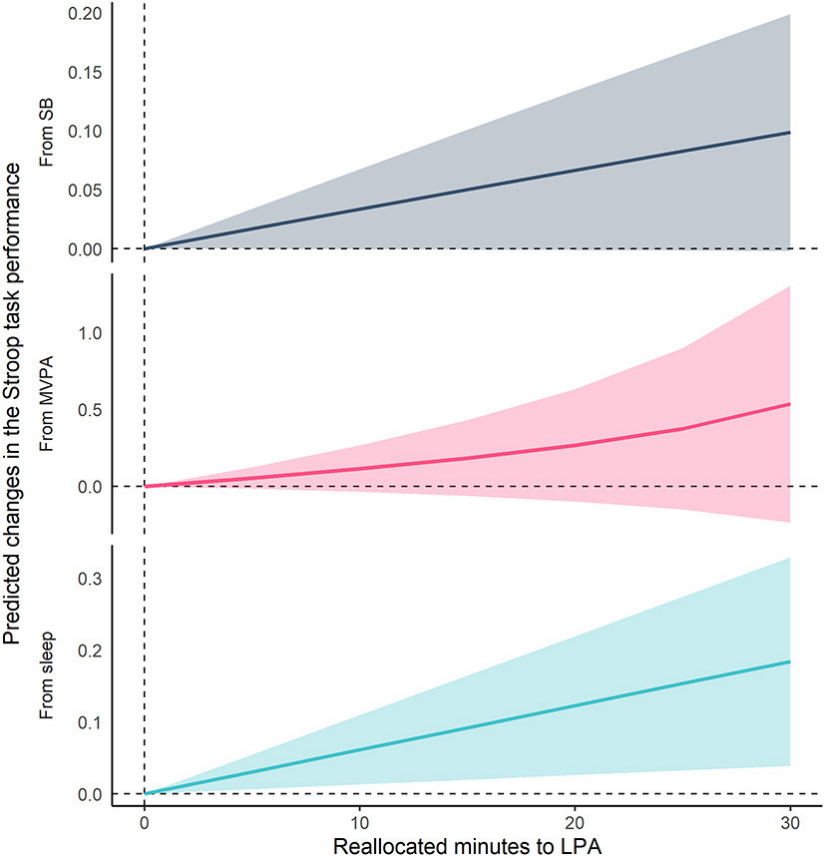
Differences in predicted changes of Stroop task performance when fixed amounts of time were reallocated from one movement behavior to LPA while keeping the remaining components constant at compositional means. The analysis was based on Model 2. SB, sedentary behavior; LPA, light-intensity physical activity; MVPA, moderate- to vigorous-intensity physical activity.

**Figure 3:**
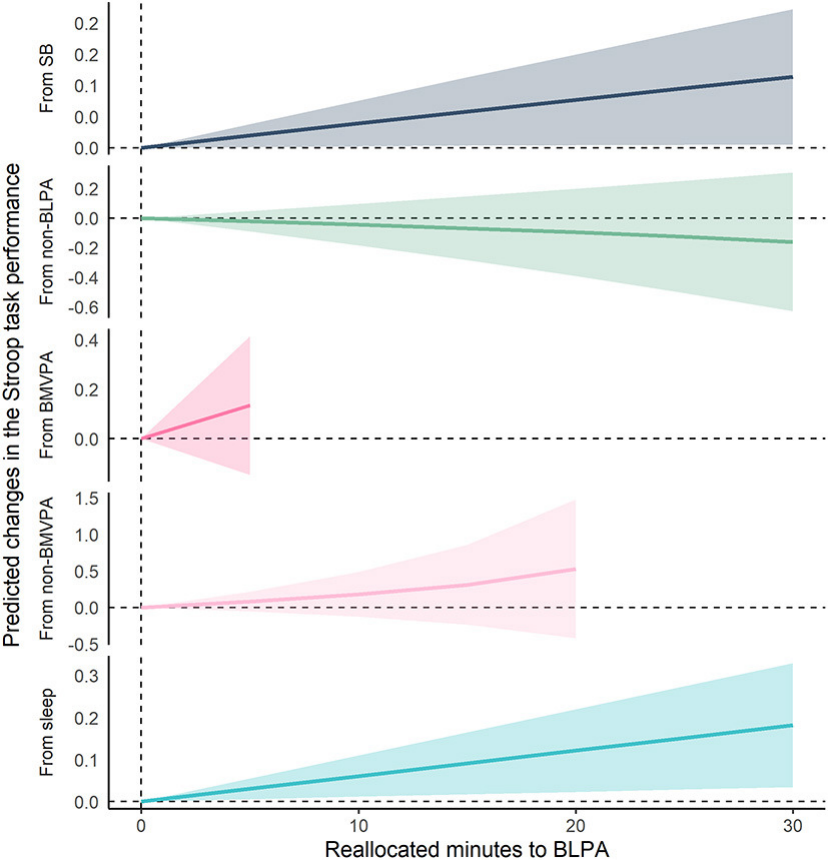
Differences in predicted changes of Stroop task performance when fixed amounts of time were reallocated from one movement behavior to BLPA while keeping the remaining components constant at compositional means. The analysis was based on Model 2. SB, sedentary behavior; LPA, light-intensity physical activity; BLPA, bouted LPA; MVPA, moderate- to vigorous-intensity physical activity; BMVPA, bouted MVPA.

Figures 2, 3 show the results of compositional isotemporal substitution (Supplementary Table 4 for the full results). We found that a longer time reallocation from SB or sleep to LPA was associated with better Stroop task performance. In particular, reallocating 30 min/day from SB or sleep to LPA was associated with better Stroop task by 0.099 (95% CI = −0.002, 0.199; corresponding to a 4.8% increase) and 0.184 (95% CI = 0.039, 0.329; corresponding to a 9.1% increase), respectively. We also confirmed that similar effect sizes, which showed the associations of time-reallocation from SB or sleep to BLPA, namely 30 min/day from SB or sleep to BLPA, were associated with better Stroop performance by 0.114 (95% CI = 0.005, 0.222; corresponding to a 5.6% increase) and 0.182 (95% CI = 0.034, 0.330; corresponding to an 8.9% increase), respectively.

## 4. Discussion

This study examined the association of accelerometer-measured PA with computer-based executive function in older adults while properly considering the co-dependent nature of daily time use using the CoDA approach. Our results indicate that longer time spent in LPA, but not MVPA, was associated with higher inhibitory control in executive function. Interestingly, the association was stronger for LPA lasting at least 10 min than for sporadic LPA. Moreover, we found that time reallocation from SB or sleep to LPA was associated with better inhibitory control. These findings provide useful information for developing healthcare programs to maintain inhibitory control in older adults.

Only one cross-sectional study has examined the association between 24-h activity duration, including LPA, and executive function in older adults (Fanning et al., 2017). Fanning et al. (2017) assessed the visuospatial domain of working memory and task-switching ability for executive function but did not evaluate inhibitory control. To the best of our knowledge, this is the first study to report a significant association between inhibitory control and device-measured LPA in older adults, with appropriate consideration of the co-dependency of daily time use. Recently, Volders et al. (2021) examined the association between longitudinal changes in PA measured by Actigraph and several cognitive performances in older adults with at least one chronic illness. They found that the change in LPA from baseline to the 6-month follow-up was significantly associated with the change in stop-signal task performance (inhibitory control) over 6 months. This supports our findings that daily LPA is positively associated with inhibitory control in older adults.

In this study, the association between LPA and inhibitory control was more pronounced for long bouts (bouts of ≥10 min) than for short bouts (bouts of < 10 min). Our results are supported by the findings of experimental studies reporting that the positive effect of single bout PA on the Stroop task (inhibitory control) was only observed when it continued for at least a few minutes (Byun et al., 2014; Chang et al., 2019a). In contrast, an observational study reported that, regardless of its bout length, total PA was not significantly associated with flanker incongruent RT (inhibitory control) (Wanigatunga et al., 2018). Considering that the majority of total PA consists of LPA (our study: ~90% of the total time in PA), the LPA in this study and the total PA in previous study would include similar activities; however, their results were inconsistent. We believe that the inconsistency in the results between the present and previous studies might be partially explained by the difference(s) in the analytical method (CoDA vs. non-CoDA) or the assessment method of inhibitory control (Stroop vs. Flanker).

The present study did not find an association between time spent in LPA and working memory or cognitive flexibility assessed by BIS of 2-back condition and BIS of global switching cost, respectively. The results did not change when AC and RT were analyzed respectively as outcomes (Supplementary Figure 1). To date, inconsistent results regarding the relationship between LPA and working memory have been reported (Umegaki et al., 2018; Gerten et al., 2021; Gothe, 2021). Our results were consistent with those of studies that reported no significant relationship between LPA and working memory assessed using the Digit Span task in community-dwelling older adults (Umegaki et al., 2018; Gerten et al., 2021). Regarding cognitive flexibility, previous studies on community-dwelling older adults reported that device-measured LPA was associated with better trail-making test performance (Johnson et al., 2016; Umegaki et al., 2018; Gerten et al., 2021; Gothe, 2021). The task-switching task used in this study did not require as much motor control or visual search as the trail-making test (Gaudino et al., 1995; Arbuthnott and Frank, 2000). Therefore, differences in assessment methods could explain the inconsistency between the results of this study and those of previous studies.

This study showed little evidence that MVPA was related to better executive function performance. Instead, it tended to be associated with lower performance. This differs from the results of a meta-analysis, which showed that moderate or vigorous exercise intervention was effective for all sub-components of executive functions (Chen et al., 2020). Observational studies using non-compositional isotemporal substitution also showed that time substitution from SB to MVPA was associated with better cognitive flexibility assessed by an RT of a task-switching task (Fanning et al., 2017), and working memory assessed by the accuracy of a spatial working memory task (Fanning et al., 2017) or score of a digit symbol substitution task (Wei et al., 2021). In the present study, the null/marginally detrimental association of the MVPA was still confirmed even when the analysis was performed using AC and RT instead of BIS as the index for executive function (Supplementary Figure 1). From a perspective other than the evaluation method for the outcome, the following points could be potential explanations for the discrepancies in findings between the present study and previous studies. First, one study reported that predicted METs using the accelerometer used in this study tend to underestimate the actual METs in the elderly, particularly during higher-intensity activities (Nagayoshi et al., 2019). Therefore, the possibility cannot be ruled out that MVPA was misclassified as LPA in this study. Second, a meta-analysis has shown that female participants experience the most beneficial effects on cognitive function with light- to moderate-intensity exercise interventions (Ludyga et al., 2020). In contrast, male participants experience further improvements with higher-intensity exercise interventions designed to increase the intensity gradually. In this study, the fact that female comprised the majority of participants (82.9%) may contribute to the favorable association of LPA, but not MVPA, with executive function. Finally, our study might not assess habitual MVPA in the participants because of the coronavirus infectious disease (COVID-19) pandemic. The PA assessment in this study was conducted in August 2021 and January 2022, when the state of emergency (July 12 to September 30, 2021) and quasi-state of emergency (January 21 to March 21. 2022) were implemented as an anti-pandemic measure in Tokyo. Under these measures, people were strongly advised to refrain from going out and meeting friends daily. During the COVID-19 pandemic, many of the community's exercise salons were closed to prevent the spread of COVID-19, and the total PA in older adults was reported to have decreased in Japan (Yamada et al., 2021). The participants in this study showed less average MVPA than Japanese older adults before the COVID-19 epidemic (36.5 min vs. the previous study: 43.6 min in men and 46.3 min in women) (Amagasa et al., 2021). In this study population, even older individuals who were physically active and maintained higher executive function before the COVID-19 outbreak may have had a temporary decrease in MVPA due to the pandemic at the time of measurement. Consequently, this study's temporal changes in PA due to the COVID-19 pandemic may have weakened the association between MVPA and executive function.

In CoDA, regression coefficients for the time spent in PA converted to log-ratios are difficult to interpret directly or alone (Dumuid et al., 2018). Hence, we conducted compositional isotemporal substitution to interpret the results for a more relevant message to public health. The results showed that replacing SB and sleep with LPA for 30 min/day was theoretically associated with increased Stroop task performance by approximately 5%-10%. This result was supported by recent systematic reviews that reported associations between too much SB and short and long sleep durations with poor cognitive performance including executive function (Lo et al., 2016; Falck et al., 2017). MVPA is often challenging for the older adult due to physical limitations and risk of injury (Schutzer and Graves, 2004; Bethancourt et al., 2014). On the other hand, LPA, such as light housework and slow walking, can be safely performed in various daily living situations even by older adults with limited physical function compared to MVPA. Therefore, the present study suggests that reallocating time spent in SB or sleep to LPA might be a feasible and sustainable strategy to manage inhibitory control in later life.

Human and animal experimental studies suggest that the mechanisms underlying PA-induced improvements in cognitive function, including executive function, could be the upregulation of BDNF, IGF-1, and VEGF (Cotman et al., 2007; Soya et al., 2007), upregulation of neurogenesis (Inoue et al., 2015), increases in brain volume (Erickson et al., 2014) and functional connectivity (Voss et al., 2010), and increases in anti-inflammatory effects (Di Benedetto et al., 2017). On the other hand, a previous study suggested that social participation (Kelly et al., 2017), mainly included in LPA, is associated with better executive function. Given this relationship, it is possible that older adults with high LPA in this study have higher social activity, which has a positive impact on executive function. However, since our regression model included variables for the frequency of social participation, the results of this study partially excluded the effect of social activity. Therefore, although it is difficult to infer the mechanisms from this study, the positive association between LPA and inhibitory control may be explained by the physiological responses to LPA.

Our study has several strengths. This is the first study to examine the association between accelerometer-measured PA and computer-based executive function in older adults, considering the co-dependent nature of 24-h movement behavior. In addition, taking advantage of the accelerometer for PA assessment, we examined the relationship between PA bouts and executive function. However, the present study has some limitations. First, as this study had a cross-sectional design, we cannot infer the cause of our results. However, a recent systematic review reported that exercise training interventions effectively improved executive function in older adults (Chen et al., 2020). This finding supports our results. Second, our study may have contained measurement errors in assessing movement behaviors. In this study, data from the questionnaire (sleep) and device (PA and SB) were integrated and standardized across 24 h for all participants' behaviors. However, this method does not eliminate the problems of measurement errors and information bias of time spent in the behaviors (e.g., underestimation of SB and LPA at periods when accelerometers are often not worn, such as just after waking or just before going to bed). Although this method is a common technique used in previous studies, future research needs to evaluate the full 24-h behavior using the device alone. In addition, PA was assessed using a hip-mounted accelerometer, which cannot completely detect postural changes. These measurement errors may cause the misclassification of behaviors. Third, we did not evaluate sleep quality or efficiency, which may be related to cognitive function (Gildner et al., 2014). Therefore, it is necessary to examine the association between PA and the quality and efficiency of sleep for a better understanding of sleep. Fourth, as this study's sample size was relatively small, the results should be considered with caution. Fifth, we cannot rule out the possibility that our results were affected by unmeasured confounders, even though various factors used as confounders in previous studies were adjusted for in this study. Finally, the external validity of this study is not clear. Because this study was based on older adults who voluntarily participated in an exercise intervention study and had a relatively small sample, it may lack representativeness. Thus, the generalizability and transformability of the results of this study should be carefully considered.

In conclusion, our study examined the association between PA and the range of executive functions in older adults while properly considering the co-dependent nature of daily time use using the CoDA. We found that a longer time spent in LPA (relative to the remaining behaviors) was associated with better inhibitory control in older adults. Specifically, this association was more pronounced for LPA occurring over at least 10 consecutive minutes than for sporadic LPA. Moreover, time reallocation from SB or sleep to LPA was associated with better inhibitory control. These findings suggest that reducing the time spent in SB or sleep and increasing the time spent in LPA, especially in BLPA, might be an important strategy for managing inhibitory control in later life. Large longitudinal and interventional studies are needed in the future to reveal the causality and underlying mechanisms.

## Data availability statement

The datasets presented in this article are not readily available because the data is still being used for scientific research. Requests to access the datasets should be directed to KH, k-hyodo@my-zaidan.or.jp.

## Ethics statement

The studies involving human participants were reviewed and approved by the Ethics Committee of the Physical Fitness Research Institute of Meiji Yasuda Life Foundation of Health and Welfare. The patients/participants provided their written informed consent to participate in this study.

## Author contributions

KH contributed to the conceptualization, data collection, data analysis, and writing of the first draft of the manuscript. NK contributed to the conceptualization, methodological development, data analysis, and writing of the first draft of this manuscript. AU contributed to the data collection and writing of the first draft of this manuscript. DY and YW contributed to the data collection and manuscript revision. TN and SN contributed to the data collection. YK contributed to the manuscript revision. TA contributed to data analysis and revision of the manuscript. All the authors approved the submitted version.
