# Supplementary material for: Association between intensity or accumulating pattern of physical activity and executive function in community-dwelling older adults: A cross-sectional study with compositional data analysis
Source: Front Hum Neurosci. 2023 Jan 25;16:1018087. doi: 10.3389/fnhum.2022.1018087 (PMC9905631; doi:10.3389/fnhum.2022.1018087)
Supplement: Supplementary file 5 [file Image_1.pdf]

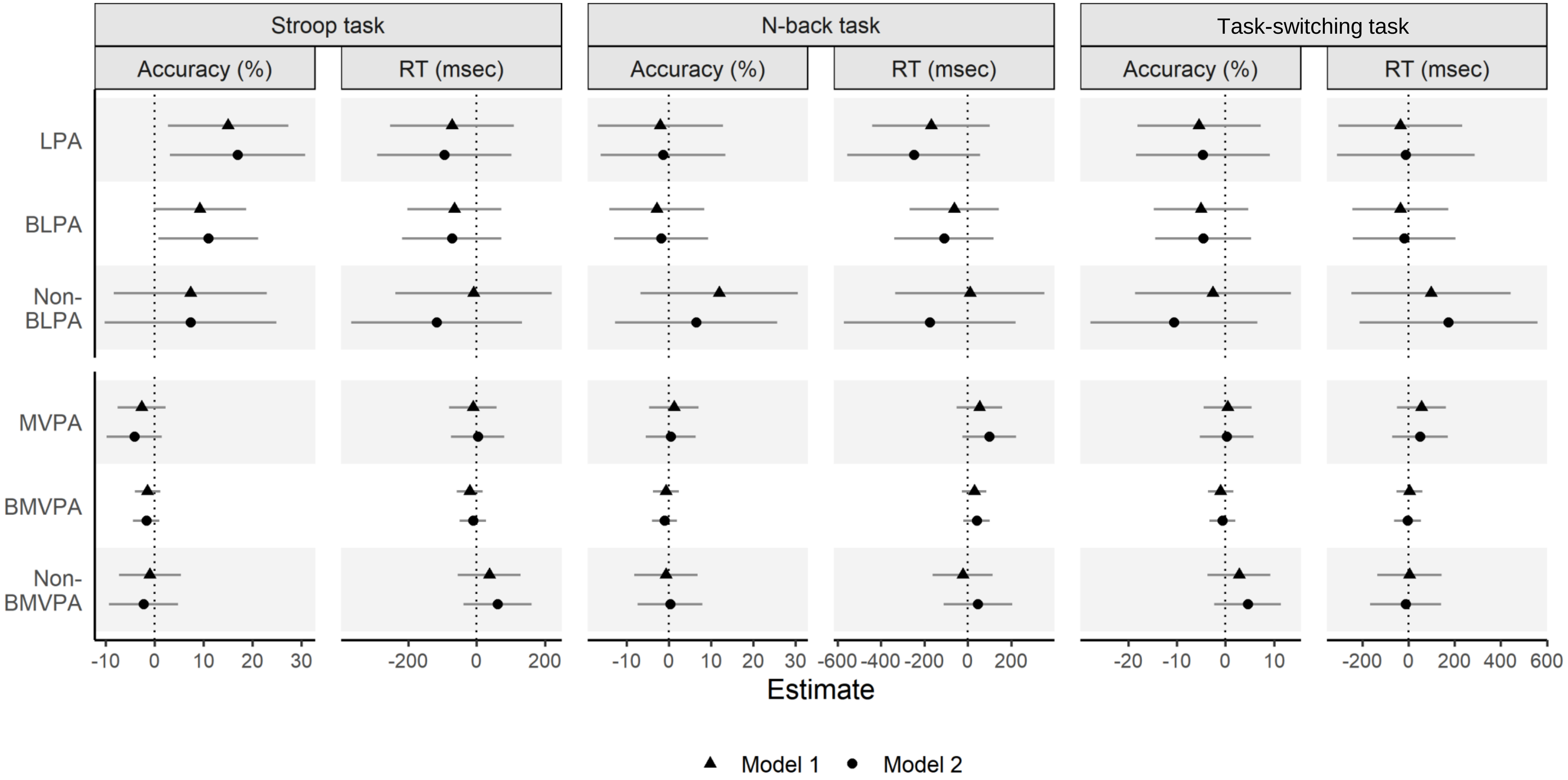

Supplementary Figure 1. Associations between physical activity and accuracy and RT for each cognitive task. Model 1 was adjusted for age, sex, and education year. Model 2 was further adjusted for subjective economic status, living arrangement, frequency of social participation, depressive symptoms, and clinical history (hypertension, diabetes, and heart disease). LPA, light-intensity physical activity; BLPA, bouted LPA; MVPA, moderate- to vigorous-intensity physical activity; BMVPA, bouted MVPA; RT, reaction time. Stroop interference, 2-back condition, and global switching cost were used for evaluating Stroop task, N-back task, and task-switching task performance, respectively.
